# Knowledge, attitude, and practice among medical students in gaza strip towards voluntary blood donation: a cross-sectional study

**DOI:** 10.1186/s12913-023-10338-5

**Published:** 2023-12-01

**Authors:** Muath Alsarafandi, Abd Al-Karim Sammour, Younis Elijla, Belal Aldabbour, Deema Muhaisen, Heba Abu Shiha, Abdalmajid Alasttal, Nour Dalloul, Anas Abuhaiba

**Affiliations:** Faculty of Medicine, Islamic University of Gaza, P.O. Box 108, Gaza, State of Palestine

**Keywords:** Donor, Blood, Knowledge, Attitude, Practice

## Abstract

**Background:**

A major component of emergency medical care is blood. The Gaza Strip has faced repeated wars over the last few years, emphasizing the importance of blood donation even more. This study aims to assess medical students’ knowledge, attitudes, and practices regarding voluntary blood donation in Gaza (VBD).

**Methods:**

This cross-sectional study used stratified sampling method to survey medical students at Gaza’s two medical schools, Al-Azhar and Islamic Universities, between March and April 2022. A 35-item self-administered questionnaire with four sections: demographics, knowledge, attitude, and practice, was used. The data were statistically analyzed using SPSS version 25.

**Results:**

A total of 329 students were surveyed (response rate of 89.6%). The median age was 20 (IQR = 3). Males made up 44.7% of the sample. Overall, 54.7% were found to have good knowledge about VBD, 68.1% did not know the time-to-wait between each whole blood donation, and in terms of blood donation criteria, only 30.7%, 25.2% were aware of the appropriate age and weight for donating. Moreover, school was the source of most information (66.6%). Meanwhile, 73.3% of participants expressed a positive attitude toward VBD. Only (17,6%) did not show a willingness to donate blood regardless of their relationship with the recipient. The vast majority (83.3%) had never donated blood before, and 12.5% had no plans to do so in the future. The two most common reasons for this were the lack of opportunity and health issues (31.0%, 11.9%, respectively).

**Conclusion:**

The sampled medical students had a positive attitude toward VBD, but there were deficiencies in their knowledge of blood donation criteria, and most had not donated blood. Adequate awareness campaigns are required to increase awareness about this universally and locally important subject.

**Supplementary Information:**

The online version contains supplementary material available at 10.1186/s12913-023-10338-5.

## Background

Blood transfusion is an indispensable component of modern healthcare. It saves countless lives in emergencies and improves the quality of life of millions of patients with various chronic conditions such as thalassemia, hemophilia, or even rarer diseases such as chronic demyelinating polyneuropathy (CIDP) [1]. Unfortunately, blood and blood components are finite. In 2017, global blood needs were estimated at 304,711,244 blood product units, while the global blood supply was approximately 272,270,243 units [2]. However, the global issue of limited blood supply is unequally distributed, with a larger supply gap in low-income and middle-income countries. It is believed that the poorer four-fifths of the global population has access to only one-fifth of the global blood supply [3].

Voluntary blood donation (VBD) is the non-remunerated giving of blood, plasma, or cellular components on one’s free permission without receiving payment [1]. It is the foundation of a sustainable and safe blood supply. The Lancet Commission on Global Surgery recommends an annual supply of at least 15 blood units per 1000 population [4]. Nonetheless, WHO data indicate that the median blood donation rate is three to ten times lower in low-income and middle-income countries than in high-income nations, reaching lower than four donations per 1000 people in some low-income countries [5]. In conflict areas, ensuring the safety and availability of blood and blood components is even more challenging and complex [6].

The Ministry of Health (MoH) is the main provider of blood transfusion services in Palestine [7]. In 2021, 46.2% of blood donors in the West Bank were voluntary, while 47.2% were for family and relatives (replacement donations). Similarly, VBDs accounted for 48.8% of the blood supply in the Gaza Strip during the same year, while the remaining were mostly replacement donations [7]. The Gaza Strip is particularly vulnerable to disruptions in the availability of blood units owing to repeated military conflicts such as the recent major conflict in 2021, with tens of thousands of casualties during the past two decades [8]. The Ministry of Health in Gaza collects blood through nine governmental blood banks, while the non-governmental Blood Bank Association operates three other blood banks [9]. MoH annual report in 2020 indicates that the Ministry’s Gaza blood banks collected 190,941 blood units between 2015 and 2020, while 366,097 were supplied over the same period [9]. Although the report does not explain how the gap was bridged, the numbers still reflect a staggering deficit between needs and actual supply in the conflict-torn enclave.

Studying the population’s knowledge, attitude, and practice (KAP) towards VBD is necessary to assess public perceptions and identify physical and cultural barriers to practicing VBD. KAP surveys also can serve as a reference to gauge the adequacy and effectiveness of interventions [10]. In our literature review, we have identified no previous studies that have evaluated the KAP towards VBD in Gaza. In a few years, current medical students will represent the core of the healthcare workforce. Medical curricula should ensure that they are capable of communicating correct information to their communities and spreading awareness; therefore, this research aims to assess medical students’ KAP towards VBD as a first step towards broader similar surveys.

## Methods

### Study design

This study used a descriptive, cross-sectional design to assess KAP towards VBD among participants.

### Setting

The study was conducted at Gaza’s two medical faculties at The Islamic University of Gaza (IUG) and Al-Azhar University (AU) between March and April 2022. Before conducting the study, written ethical approval was obtained from the Deanships of Scientific Research and Development at both universities. Verbal consent was obtained from participants before survey completion. Confidentiality was guaranteed to all participants and maintained throughout the data analysis. The study was conducted in accordance with all relevant guidelines and regulations.

### Participants

The study included medical students enrolled in basic or clinical studies who consented to fill out the questionnaire. Students absent during the first round of data collection were offered to participate in follow-up rounds. The study excluded medical students who refused to participate.

### Questionnaire

The study team constructed a questionnaire based on a literature review of previous similar studies [11–13]. The questionnaire included 35 items divided into four sections (supplementary material). The first section included the sociodemographic data of participants. The second Sect. (20 questions) assessed participants’ knowledge in the following manner: eight questions assessed participants’ general knowledge about blood donation, seven assessed participants’ knowledge regarding criteria for the blood donor, and 5 assessed participants’ knowledge regarding transmissible transfusion diseases. The third Sect. (7 questions) assessed participants’ attitudes, and the fourth Sect. (8 questions) assessed participants’ practice toward VBD. The questionnaire was presented in English since it was the language of teaching in both medical faculties. Public health experts from the IUG evaluated the questionnaire’s face, content, and convergent validity. The questionnaire was then piloted for acceptability and consistency with 20 medical students conveniently sampled from different levels. After the pilot testing, a few minor adjustments were required. Data from the pilot study was discarded.

### Sample size

The single population proportion formula was used to determine the sample size. The marginal error was set at 5% (d = 0.05) as the CI = 95% (Zα/2)2 = 1.96. There are no previous studies in the Gaza Strip about this topic, so the expected frequency P was set to 50%.

$$n=\frac{{\left(\frac{\text{Z}{\upalpha }}{2}\right)}^{2}\text{*}\text{p}\left(1 - \text{p}\right)}{{\text{d}}^{2}}=\frac{{\left(1.96\right)}^{2}\text{*}0.5\left(1 - 0.5\right)}{{0.05}^{2}}=384.16$$

To calculate our sample size, the Finite Sample Size equation was used (Total population is 2215):

$$n=\frac{384.16}{1+\frac{384.16-1}{2215}}=327$$

An additional 12% of the calculated size was added to account for potential non-responders (327 × 12% = 40); thus, the final sample size before stratification became 367.

To ensure equal representation of all categories, students were stratified according to university and study level, depending on their respective population sizes.

### Statistical analysis

The medical students who scored ten or more correct answers out of 20 questions in the second section were considered to have adequate knowledge about VBD, while scores below ten were regarded as insufficient knowledge. In terms of attitude, a score of six or seven reflected a good attitude. Meanwhile, practice questions were presented as percentages (not assembled in a score). These measurements resulted from the normality characteristics of data distribution, either 25–75 quartiles or median. Data were analyzed using the statistical package for social sciences (SPSS) version 25 (SPSS Inc., Chicago, IL, USA). Data analysis provided frequency tables for variables. Kolmogorov-Smirnov test was used to assess the sample distribution’s normality. Kruskal-Wallis, Mann-Whitney, and Chi-Square tests were used to determine the relationship between the dependent variables (knowledge, attitude, and practice) and the independent categorical variables of the sociodemographic data. The multinomial logistic regression was used to predict the relationship between the cohort characteristics and KAP domains. Statistical significance was set at *p* values of less than 0.05.

## Results

### Sociodemographic characteristics of the cohort

The sample was comprised of 329 medical students from Gaza’s two medical schools who filled out the questionnaire (53.2% from Al-Azhar and 46.8% from IUG). The response rate was 89.6%. Females represented 55.3% of participants. The largest two groups were the first and second levels (34.0% and 20.4%, respectively) (Table 1).

**Table 1:** Demographic characteristics of the participants

Demographic characteristics	n	Percent
**Gender**		
Females	182	55.3%
Males	147	44.7%
**University**		
Al-Azhar University of Gaza	175	53.2%
Islamic University of Gaza	154	46.8%
**Academic Level**		
First Level	112	34.0%
Second Level	67	20.4%
Third Level	43	13.1%
Fourth Level	40	12.2%
Fifth Level	39	11.9%
Sixth Level	28	8.5%
Total	329	100.0%

### Knowledge about VBD

Overall, 180 participants (54.7%) demonstrated good knowledge about VBD, and 220 (66.9%) were familiar with their blood type. Regarding general knowledge about VBD, 105 (31.9%) knew the healthy frequency for blood donation. Also, 145 (44.1%) of the participants correctly identified the volume of blood donated, while 158 (48%) recognized the duration of the blood collection process. On the other hand, only 83 participants (25.2%) answered the minimum weight of a blood donor correctly, while 77 (23.4%) knew when postpartum mothers could start donating blood (Table 2). The Kruskal-Wallis and Mann-Whitney tests showed significant differences in knowledge levels among genders and academic levels but no significant differences between students of different universities (Table 3). Using logistic regression to determine the predictors of good knowledge showed that first-level students are more likely to demonstrate low knowledge regarding VBD than others, with an odds ratio of (0.280) (Table 4).

**Table 2:** Participant’s responses to knowledge of VBD

	VBD Knowledge Items	Correct Answer	n	Percent (correctly answered)
**General knowledge about blood donation**	Do you know your blood group	Yes	220	66.9%
	How frequently can a person donate blood?	8 Weeks	105	31.9%
	What is the volume of blood collected during each blood donation?	< 500 ml	145	44.1%
	What is the duration of a donation process?	20–60 m	158	48.0%
	Is screening of blood necessary before donation?	Yes	279	84.8%
	When is the blood donation day?	14th June	111	33.7%
	Which blood group is considered the universal donor?	O -	252	76.6%
	Which blood group is considered the universal recipient?	AB +	258	78.4%
**Knowledge regarding criteria for a blood donor**	What is the minimum age to start blood donation?	16y	101	30.7%
	What is the minimum weight for blood donation?	55 Kg	83	25.2%
	What is the minimum hemoglobin level required to donate blood?	Men (13)	144	43.8%
		Women (12.5)	210	63.8%
	What is the required blood pressure at the time of blood donation?	Highest (< 180\100)	201	61.1%
		Lowest (> 90\50)	74	22.5%
	What is the minimum Duration between delivery of baby and blood donation?	6 weeks	77	23.4%
	Is fever a contraindication to blood donation?	Yes	179	54.4%
	Are contraceptive pills contraindication to blood donation?	No	122	37.1%
**Transfusion transmissible diseases**	Can HIV be transmitted via blood donation?	Yes	254	77.2%
	Can Hepatitis B be transmitted via blood donation?	Yes	259	78.7%
	Can Syphilis be transmitted via blood donation?	Yes	145	44.1%
	Can Giardia be transmitted via blood donation?	No	160	48.6%
	What are your sources of information about blood donation? ^*^	School-College	219	66.6%
		Mass Media	70	21.3%
		Blood donation camp	26	7.9%
		Friends \ parents	61	18.5%
		Have no information	33	10.0%

^*^ Can apply more than one

**Table 3:** Association between demographic characteristics and KAP components

Demographic characteristics	n	Knowledge score Mean (SD)	*P*-value	Attitude score Mean (SD)	*P*-value	Practice (Donation History) Percent (Yes)	*P*-value
**Gender**			**0.013** ^a^		**0.023** ^a^		**0.000** ^c^
Females	182	10.16 (3.965)		6.12 (1.073)		7.7%	
Males	147	9.01 (3.361)		5.73 (1.412)		27.9%	
**University**			0.278 ^a^		0.913 ^a^		0.416 ^c^
Al-Azhar University of Gaza	175	9.39 (4.178)		5.90 (1.338)		18.3%	
Islamic University of Gaza	154	9.94 (3.174)		5.99 (1.143)		14.9%	
**Academic Level**			**0.000** ^b^		**0.001** ^b^		0.884 ^c^
First Level	99	7.96 (4.120)		5.63 (1.440)		15.2%	
Second Level	59	9.00 (3.257)		5.81 (1.258)		17.9%	
Third Level	37	11.07 (2.576)		6.28 (1.076)		14.0%	
Fourth Level	36	11.15 (3.505)		6.08 (1.047)		15.0%	
Fifth Level	32	12.08 (3.064)		6.15 (1.014)		23.1%	
Sixth Level	28	10.21 (2.299)		6.54 0.793)		17.9%	

^a^ Mann-Whitney Test, ^b^ Kruskal Wallis Test, ^c^ Chi-Square Tests, *P* < 0.05

**Table 4:** Logistic regression analysis of sociodemographic predictors of adequate knowledge towards VBD

Demographic characteristics	n	Odds ratio	CI 95 (lower bond-upper bond)	*p* value
**Gender**				
Females	182	1.194	0.748-1.908	0.458
Males	147	REF	REF	REF
**University**				
Al-Azhar University of Gaza	175	1.100	0.682-1.775	0.696
Islamic University of Gaza	154	REF	REF	REF*
**Academic Level**				
First Level	112	0.280	0.117-0.670	**0.004**
Second Level	67	0.574	0.230 − 1.430	0.233
Third Level	43	1.656	0.588-4.666	0.340
Fourth Level	40	1.328	0.474 − 3.720	0.590
Fifth Level	39	1.892	0.645-5.548	0.246
Sixth Level	28	REF	REF	REF

R^2^ = 0.117

*Reference

### Attitudes towards VBD

The majority of participants demonstrated good attitudes (241, 73.3%). For instance, 318 (96.7%) participants believed that blood donation is noble, and 296 (90%) reported that voluntary blood donation is the best source to obtain blood. Regarding whom they will donate to, the majority (293, 89.1%) said they would donate blood to a relative, while 271 (82.4%) would also donate blood to non-relatives, and 238 (72.3%) stated they would donate blood regardless of the recipient’s religion. Most participants (254, 77.2%) do not expect any rewards for donating (Table 5). There was a statistically significant difference in attitude depending on gender and academic level (Table 3). Also, being a female was a statistically significant predictor of demonstrating good attitudes, with an odds ratio of (2.062) (Table 6).

**Table 5:** participant’s attitude towards VBD

VBD Attitude items	Favorable attitude	Participants	Percent
Is blood donation a good and noble act?	Yes	318	96.7%
What is your attitude towards blood donation?	Agree	285	86.6%
What do you think is best source of blood donors?	Voluntary	296	90%
Are you willing to donate blood to your relative?	Yes	293	89.1%
Are you willing to donate blood to Anyone?	Yes	271	82.4%
Will you donate blood without knowing the religion of the recipient?	Yes	238	72.3%
Do you expect any reward for blood donation?	No	254	77.2%

**Table 6:** Logistic regression analysis of sociodemographic predictors of adequate attitude towards VBD

Demographic characteristics	n	Odds ratio	CI 95 (lower bond-upper bond)	*p* value
**Gender**				
Females	182	2.062	1.243–3.421	**0.005**
Males	147	REF*	REF	REF
**University**				
Al-Azhar University of Gaza	175	0.448	0.727-2.053	0.263
Islamic University of Gaza	154	REF	REF	REF
**Academic Level**				
First Level	112	0.220	0.061-0.785	0.247
Second Level	67	0.286	0.077-1.070	0.231
Third Level	43	0.673	0.157 − 2.890	0.906
Fourth Level	40	0.384	0.094-1.569	0.244
Fifth Level	39	0.366	0.089-1.496	0.472
Sixth Level	28	REF	REF	REF

R^2^ = 0.056

*Reference

### Practice of VBD

Of 329 participants, 55 (16.7%) had donated blood before, with 12 (3.6%) donating more than once. Most of the donors (41 out of 55) donated blood voluntarily, and 51 out of 55 were satisfied after VBD. The top two reasons for not donating were having no opportunity to donate blood or suffering from health problems (31.0% and 11.9%, respectively) (Table 7). Donation practice was statistically different between males and females (Table 3).

**Table 7:** Participant’s responses on practice toward VBD

VBD practice items	Practice	Participants	Percent
Have you donated blood before?	Yes	55	16.7%
How many times you have donated blood? ^*^	None	274	83.3%
	Once	43	13.1%
	Twice or more	12	3.6%
Why did you donate blood? ^*^	Relative needed blood	8	14.8%
	Non-relative needed blood	5	9.3%
	Voluntarily	41	75.9%
Are you satisfied after donating blood? ^*^	Yes	51	92.7%
Are you willing to donate blood in future?	Yes	288	87.5%
Have you received blood?	Yes	21	6.4%
What are the reasons for not donating so far? ^**^	Nonspecific reason	130	39.5%
	Fear	35	10.6%
	Parenteral pressure	4	1.2%
	No awareness	20	6.1%
	No opportunity	102	31.0%
	Health Problems	39	11.9%

^*^ For donors only, ^**^ for non-donors only

## Discussion

A timely and dependable access to blood and blood components is essential to modern healthcare. Forecasts from different high-income countries predict a rising imbalance between blood supply and demand, owing primarily to the aging of the population [14, 15]. Lower-income countries, on the other hand, are presently struggling with blood supply, making the future even more worrisome [5, 16]. Additionally, the stringent safety and storage requirements and the short life of donated blood further stress healthcare systems in unstable and poor regions. The present study evaluated the knowledge, attitude, and practice of VBD among medical students in the Gaza Strip, which is a low-income area marred by chronic instability and repeated military conflicts. The study showed that medical students possessed acceptable levels of good knowledge and appropriate attitudes that are comparable to healthcare workers surveyed in other countries, but the practice of blood donation was still below the recommended global rate and that needed in a war-prone enclave.

Regular VBD is the safest and most reliable method of securing blood needed for the community [1, 16]. Voluntary blood donors are driven by a sense of moral and social responsibility and are therefore perceived to be free from social, financial, or medical pressures to donate. Replacement donations by relatives of patients requiring blood transfusions may present an alternative that helps maintain blood bank stocks; however, they frequently result in delayed care and may not be sufficient in emergency or mass casualty situations, such as in areas of military conflicts. Also, the pressure on replacement donors may cause some donors to conceal relevant information about their health, ultimately impacting the safety of collected blood [1, 5]. On the other hand, governments are responsible for ensuring a transparent, integrated, effective, and safe blood banking system and providing the needed legislation and infrastructure [16]. Thus, the relative abundance of voluntary blood donors in high-income countries is likely due to interacting social, cultural, and infrastructure factors.

Appropriate knowledge about the blood donation process is essential to promoting the VBD culture. In our study, 54.7% of participants demonstrated good knowledge about VBD. This percentage is similar to the findings of a study among 598 Saudi health professions students, where 60.2% of participants had good knowledge. Meanwhile, 48.2% of participants in another study among 255 Ethiopian health science students showed good knowledge [17, 18]. In all of these countries, the numbers indicate significant knowledge gaps that need to be bridged. The fact that the studies were conducted among healthcare students also hints that other community sectors might demonstrate even lower knowledge levels.

Safety is a critical aspect of the blood collection process. The Palestinian MoH currently mandates testing for the Hepatitis B virus (HBV), Hepatitis C virus (HCV), and HIV (AIDS). Also, the syphilis test is performed for fresh transfusion units and platelets units [7]. In our cohort, 77.2%, 78.7%, and 44.1% of students were aware of the risk of HIV, HBV, and syphilis, respectively, being transmissible via blood. These percentages are lower than those reported by a study among 235 medical students from North India, where awareness about these potential infections was 99%, 97%, and 57%, and to some extent similar to the results of a Nigerian survey of a university hospital staff, among whom the percentages were 91.4%, 69.9%, and 27.0% respectively [19, 20]. Meanwhile, like our study, 75.7% of participants in the previously cited Ethiopian study identified HBV as a potentially transmissible disease [18]. These numbers reflect an additional, vital area where knowledge improvement is needed, especially since our participants will in a few years become health service providers. Nonetheless, this gap will likely diminish as students transition into higher academic levels. For instance, knowledge scores showed a significant correlation with the academic level of participants, and only 3.4% of healthcare personnel in a recent survey from the Gaza Strip did not recognize HBV as transmissible via blood transfusion [21].

Regarding the knowledge of blood donation criteria (question 9–15 in Table 2), inadequate knowledge was apparent in most questions, even in terms of the simplest criterions such as the minimal age and weight for blood donation (correctly answered by 30.7% and 25.2% of participants, respectively). In the study from India, 95% and 84% correctly answered age and weight-related questions [19]. Regarding contraindications, 54.4% of our participants correctly identified fever as a contraindication, while 37.1% erroneously stated that oral contraceptives were a contraindication towards VBD. The general level of knowledge in this domain appears particularly defective, thus there is a need for awareness activities to promote correct information and prevent the spread of misbeliefs among this cohort of future leaders in the field.

On the other hand, the majority in our cohort demonstrated a good level of healthy attitudes towards VBD (73.2%). The previously mentioned Saudi study also reported similar findings [17]. Nonetheless, our results remain better than some other studies. For example, in a comparative study between Health Science students and Non-Health Science students from an Ethiopian university, 46.7% and 35.6%, respectively, had a favorable attitude, which is significantly lower than our findings [22]. Living in an area that has been hit by repeated major conflicts and where blood donation is a matter of national and religious reward could have contributed to spreading good attitudes.

The World Health Organization (WHO) advocates that 3–5% of the population should donate blood annually [17]. In our study, 16.7% donated blood before, and 3.6% reported donating more than once. In the previously mentioned survey of Health Science students, 12.5% had ever donated blood, while 30% of participants in the Saudi study had previously donated [17, 18]. The Saudi authors argue that religion plays a role in their remarkably high figures, citing the example of a religious ruling issued by the country’s highest religious affairs body, which advocates donating blood and is frequently placed in donor centers. The association between religiousness and a healthier attitude toward VBD was reported by other researchers as well [23]. Overall, the percentage of donors from our cohort falls within the range of previous studies. Additionally, 87.5% had the intention to donate blood, and 31% of stated “having no opportunity to donate” as a cause for not donating so far. Still, efforts are needed to recruit more voluntary blood donors and to provide the needed facilities to enable the occasional donor to become a regular voluntary blood donor.

The percentage of female blood donors is estimated at 33% globally, reaching as low as 10% in some countries [16]. In our cohort, 27.9% of male participants had donated blood before, while only 7.7% of females had donated (*p* = 0.000 )(Table 3). In the Saudi, Ethiopian, and Nigerian studies, male participants were also significantly more likely to have donated blood [17, 20, 22]. This is a serious gap in practice since it reveals that half the community needs to be more involved in VBD. However, the findings also offer a unique window for a solution where the significant pool of healthy females should be the target of well-planned and focused awareness and education campaigns, ultimately benefiting society as a whole.

Different reasons were cited as causes for not donating, for example, fear, health problems, lack of awareness, and family pressure, which is comparable to other studies [24, 25]. Importantly, some of these reasons are modifiable and should be addressed by stakeholders. In Gaza, blood donation campaigns and drives are held annually. For instance, 224 blood drives were carried out in Gaza in 2020, in which 7120 blood units were collected, indicating the public’s willingness to donate blood when given the opportunity. On the other hand, a study in 2011 stated that blood deficiency in the Gaza Strip, which was most pronounced during the 21-day-long 2008/2009 war, was due to a lack of coordination between health institutions and concerned parties [26]. Palestinians live under occupation and remain under the constant threat of military conflict. While no one can foresee the future, having solid protocols and emergency plans can mitigate many of the difficulties as they unfold. This complicated and integrated process extends from gap analysis, quality control, and benchmarking to the standardization of the blood collection process and incorporation of information technology where needed. Additionally, direct communication between the different parties involved and continuous staff training is essential.

### Strengths and limitations

This is the first survey to assess the KAP of a cohort of Palestinians from the Gaza Strip towards VBD. The strengths of this study include the large cohort size and stratified randomization method, in addition to the depth of statistical analysis. Nonetheless, the surveyed population was limited to students of medical faculties. Therefore, generalizations cannot be made. Further studies are needed to assess the factors influencing VBD in the Gaza Strip comprehensively.

## Conclusion

This study showed that knowledge and practice towards VBD are suboptimal among medical students in the Gaza Strip. Nonetheless, good attitude was prevalent, indicating a hope to increase the rates of VBD and decrease the current gap between blood supply and demand. Adequate awareness campaigns are needed to increase knowledge and awareness about this universally and locally important subject.

### Electronic supplementary material

Below is the link to the electronic supplementary material.

Supplementary Material 1

## Data Availability

The datasets used and/or analysed during the current study are available from the corresponding author upon reasonable request.
