# Supplementary material for: Knowledge, attitude, and practice among medical students in gaza strip towards voluntary blood donation: a cross-sectional study
Source: BMC Health Serv Res. 2023 Dec 1;23:1333. doi: 10.1186/s12913-023-10338-5 (PMC10691018; doi:10.1186/s12913-023-10338-5)
Supplement: Supplementary file 1 — Supplementary Material 1 [file 12913_2023_10338_MOESM1_ESM.docx]

- **Study Questionnaire**

| **Gender** | Male | Female | | **Age** |  | **University** | IUG | AUG |
| --- | --- | --- | --- | --- | --- | --- | --- | --- |
| **Level** | First | | Second | | Third | Fourth | Fifth | Sixth |

| **General knowledge about blood donation** | | | | | | | | | | |
| --- | --- | --- | --- | --- | --- | --- | --- | --- | --- | --- |
| 1 | What is Your blood group? |  | | | | | | | | |
| 2 | How frequently can a person donate blood? (Weeks) | 4 | 6 | | | 8 | | | | 10 |
| 3 | What is the volume of blood collected during each blood donation?  (ml) | < 500 | | | 500-1000 | | | | 1000 > | |
| 4 | What is the duration of a donation process? (minutes) | 10-20 | | | 20-60 | | | | 60 > | |
| 5 | Is the screening of blood necessary before donation? | Yes | | | | | No | | | |
| 6 | When is the blood donation day? | 7^th^ April | | | 16^th^ May | | | | 14^th^ June | |
| 7 | Which blood group is considered the universal donor? | O+ | | O- | | | AB+ | | | AB- |
| 8 | Which blood group is considered the universal recipient? | O+ | | O- | | | AB+ | | | AB- |
| **Knowledge regarding criteria for a blood donor** | | | | | | | | | | |
| 9 | What is the minimum age to start blood donation? | 14y | | 16y | | | 18y | | | 20y |
| 10 | What is the minimum weight for blood donation? | 35kg | | 45kg | | | 55kg | | | 65kg |
| 11 | What is the minimum hemoglobin level required to donate blood? | Men | | | 12.5 | | | 13 | | 13.5 |
|  |  | Women | | | 12.5 | | | 13 | | 13.5 |
| 12 | What is the required blood pressure at the time of blood donation? | Highest | | | <180\100 | | | | <200\120 | |
|  |  | Lowest | | | >100\60 | | | | >90\50 | |
| 13 | What is the minimum Duration between delivery of baby and blood donation? | 4 | | 6 | | | 8 | | | 10 |
| 14 | Is fever a contraindication to blood donation? | Yes | | | | | No | | | |
| 15 | Are contraceptive pills contraindication to blood donation? | Yes | | | | | No | | | |

| **Knowledge regrading transfusion transmissible diseases** | | | | | | | |
| --- | --- | --- | --- | --- | --- | --- | --- |
| 16 | Can **HIV** be transmitted via blood donation? | | | | Yes | | No |
| 17 | Can **Hepatitis B** be transmitted via blood donation? | | | | Yes | | No |
| 18 | Can **Syphilis** be transmitted via blood donation? | | | | Yes | | No |
| 19 | Can **Giardia** be transmitted via blood donation? | | | | Yes | | No |
| 20 | What are your sources of information about blood donation? | | | | | | |
| School-College | | Mass Media | Blood donation camp | Friends \ parents | | Have no information | |

| **Attitude** | | | | | | | | | | | | |
| --- | --- | --- | --- | --- | --- | --- | --- | --- | --- | --- | --- | --- |
| 21 | Is blood donation a good and noble act? | | | | | Yes | | | No | | | |
| 22 | What is your attitude towards blood donation? | | | | | Agree | Neutral | | | Disagree | | |
| 23 | What do you think is best source of blood donors? | | | | | Paid | Voluntary | | | Replacement | | |
| 24 | Are you willing to donate blood to your relative? | | | | | Yes | | | No | | | |
| 25 | Are you willing to donate blood to Anyone? | | | | | Yes | | | No | | | |
| 26 | Will you donate blood without knowing the religion of the recipient? | | | | | Yes | | | No | | | |
| 27 | Do you expect any reward for blood donation? | | | | | Yes | | | No | | | |
| **Practice** | | | | | | | | | | | | |
| 28 | Have you donated blood before? | | | | | Yes | | | No | | | |
| 29 | How many times you have donated blood? | | | | | None | | Once | | | >Twice | |
| 30 | Why did you donate blood? | | | | Relative needed blood | | | Non-relative needed blood | | | | Voluntary |
| 31 | Are you satisfied after donating blood? | | | | | Yes | | | No | | | |
| 32 | Are you willing to donate blood in future? | | | | | Yes | | | No | | | |
| 33 | Have you received blood? | | | | | Yes | | | No | | | |
| 34 | What are the reasons for not donating so far **(For non-donors only)** | | | | | | | | | | | |
| Nonspecific reason | | Fear | Parenteral pressure | No awareness | | | No opportunity | | | | | |
